# “I wasn’t ready”: abortion decision-making pathways in Ouagadougou, Burkina Faso

**DOI:** 10.1007/s00038-020-01359-6

**Published:** 2020-04-09

**Authors:** Ramatou Ouedraogo, Leigh Senderowicz, Coralie Ngbichi

**Affiliations:** 1 African Population and Health Research Center, Nairobi, Kenya; 2 Department of Global Health and Population, Harvard T.H. Chan School of Public Health, Boston, USA

**Keywords:** Abortion, Young people, Ethnography, Burkina Faso, West Africa, Decision making

## Abstract

**Objectives:**

This study explores abortion decision-making trajectories in Ouagadougou, Burkina Faso, examining the spaces for decision making that young people manage to create for themselves within restrictive policy, gender norms and other constraints.

**Methods:**

The study presents data collected from observations in three referral health facilities in Ouagadougou and interviews (with 31 young women (aged 17–25) who had sought abortions and five men (aged 20–25) whose partners had done so). Using inductive content analysis, we capture the different streams, actors and rationales in the decision-making process, as well as the pattern of negotiation.

**Results:**

Abortion decision-making trajectories are complex and affected by a range of factors including fertility desires, relationship stability and financial stability. The process can include intense periods of negotiation between intimate partners when their rationales are discordant. Constraints on women’s decision making include restrictive policy environment, coercion from partners (threats, emotional blackmail and even physical force) and pressure from people in and out of their social network.

**Conclusions:**

In a context where legal abortion is highly restricted and women’s decision-making power is constrained, the abortion decision making appears as collective, operates in an uncertain time frame, an unofficial social environment and has an unpredictable collaborative mechanism.

## Introduction

Burkina Faso is a landlocked country in the Sahel region of West Africa. Formerly a French colony, abortion in Burkina Faso is legally restricted to cases of fetal malformation, rape, incest, or when the pregnancy presents a danger to women’s health. Even in these cases where the laws make clear provision for abortion access (for rape survivors, for example), the challenges in the implementation of the related policies and lack of awareness about the laws lead to a crucial lack of access to legal abortion services (Ouedraogo and Ouattara 2013; Sombié et al. 2018; Amnesty International 2009). Out of these legal conditions, women who induce abortion and providers who deliver abortion services risk prosecutions (imprisonment and fines). In addition to the legal obstacles and health implications, abortion in Burkina Faso is also subject to social and religion stigma. A 2010 survey ranked abortion as the third most condemned practice after homosexuality and prostitution (CGD 2010). And yet, despite the social, legal and health-related hurdles, abortion remains a common practice in Burkina Faso. In 2012, the abortion rate was estimated at 25 per 1000 women aged 15–44 years (Bankole et al. 2013), resulting in an approximately 105,000 abortion per year, half of which resulted in complications (Bankole et al. 2013; Turner et al. 2016). Of these complications, approximately 40% are reported to have not benefited from any medical treatment, making abortion a leading cause of maternal death in the country (Bankole et al. 2013; Ministry of Health of Burkina Faso 2011).

Reasons for terminating pregnancy have been documented in several settings throughout Africa (Bankole et al. 1998; Chae et al. 2017; Bankole et al. 2013; Bleek 1990; Guillaume and Rossier 2018; Svanemyr and Sundby 2007). Women’s reasons include a desire to postpone/space childbearing, concerns that they are too young to become parents, are carrying a stigmatized pregnancy (premarital or adulterous relationship’ pregnancy, for example) and financial constraints, among many others. The bulk of the existing literature on decision making focuses on the driving factors (fertility intentions, norms, resources, law) as well as the decision outcomes (Coast et al. 2018; Orner et al. 2010; Orner et al. 2011; Frederico et al. 2018; Kumi-Kyereme et al. 2014; Whittaker 2002). The dynamic of the decision-making process itself—structures, sequences, time frame—has been overlooked, especially in restrictive contexts. The evidence on abortion decision making comes mostly from settings with less restrictive abortion policy environments and provides insights into the process (starting from the reactions, impact factors and tools for process), as well as the actors involved and power relationship, and how the dynamic within the process can lead to delays in seeking abortion (Harries et al. 2007; Ekstrand et al. 2009; Friedlander et al. 1984; Donati et al. 2002; Holmberg and Wahlberg 2000). In restrictive settings where legislation does not confer any decision-making authority to women (it is granted instead, in limited legal circumstances to doctors and judges), some studies have documented the key role of the partners and family members in the decision-making process (Kumi-Kyereme et al. 2014). However, they do not inform on how these actors as well as the legislation shape the process, especially the time frame, and the patterns of negotiation.

This study seeks to explore the pathways to abortion decision making in Burkina Faso. Adding to the body of literature on abortion trajectories and decision making, we examine the decision-making space (in terms of dynamic), and how young women and men navigate through a sea of gender roles, intergenerational pressures, restrictive policies and other constraints to arrive at their reproductive decisions (Coast et al. 2018; Bonnet 1988; Badini 1978; Dacher 1999; Vinel 2005; Gruénais 1985; Attané 2007). In particular, this paper focuses on young women and men who arrived at the conclusion that they were not “ready” to become parents and the process that led to such conclusion. In a context where improving young people’s sexual and reproductive health and rights is an increasingly important part of the global health agenda, this nuanced understanding of the abortion decision-making process can contribute to a broader understanding of gendered power dynamics and that ways that social norms and policy environments contribute to important lapses in health care.

## Methods

### Study site, participants and approach

This ethnography was carried out between 2011 and 2014 as part of a broader project on access to abortion and post-abortion care (PAC) services in Burkina Faso. Data were generated using observation and in-depth interviews (IDI) through a facility and network-based approach. We first observed situations of PAC services delivery in three referral health facilities all located in Burkina Faso’s capital Ouagadougou. During the immersion in these facilities, the first author was alternately a receptionist, counselor, translator and care assistant. As an assistant, for example, her role was to fill patient admission forms or support providers during manual vacuum aspiration (MVA) by helping to immobilize patients. This immersion provided an opportunity to gather information on provider–patient interactions and also obtain initials insights into the decision-making pathways (especially the concept of “not ready” used by most of the young women to justify their decision to abort).

The observations allowed us to identify our participants. In collaboration with the healthcare providers, we approached all women who were treated for pregnancy termination attempts or complications and women seeking abortion services to terminate their pregnancy. We presented the study to the potential participants and recruited those who consented to be part of the study. We identified a total of 68 women with whom we exchanged contact information before they were discharged. Later, we were able to reach and visit only 44 of these women aged 17–33. Three partners of these women agreed to participate in the study and were then included our sample.

Out of the facilities, we also identified and recruited two young women (respectively, 22 and 24 years) who had completed an abortion and four men (21–27 years) whose partners had abortion through our personal network. Friends, colleagues and relatives who were aware of this study connected us with women and men they know had experienced abortion. By recruiting some of the participants through at the community level, we wanted to have a different perspective and experiences compared to those recruited in the health facilities. In fact, women recruited through the personal network had medical abortion without complications and did not require post-abortion care.

After the selection, we conducted in-depth interviews (IDIs) in French and local languages (Moore and Dioula) with all 53 participants using a developed and tested semi-structured interview guide. All IDIs were conducted in various places depending on the participants’ preferences. Additional interviews (including informal discussions) were conducted with 20 of the participants—15 women and five men—with whom the first author developed a close relationship to enhance trust to overcome the silence and stigma surrounding abortion. As the study touches a sensitive subject, the research team wanted to have the additional interviews only with participants showing interest in developing thoroughly their experience and story.

The IDIs were conducted to get as close as possible to an informal discussion led by the priorities of the participants. We first asked general questions (about their life, family, school or work, etc.). Then, questions focused on the conception of motherhood and fatherhood, contraceptive use, the process of the decision to terminate a pregnancy, the people involved and the factors driving the decision making (norms, law, their life goals, availability of abortion services, etc.). The materials collected were rich in personal, familial, socioeconomic and romantic events which drive the person’s life before the pregnancy and toward the decision-making process. All interviewees were assigned pseudonyms names, and no identifying information was retained. This paper is drawn only from the experiences of young participants aged 17–25, giving a total of 36 (31 women and five men) out of the 53 interviewed. Their socio-demographics characteristics are summarized in Table 1.

**Table 1:** Socio-demographic characteristics of participants, Burkina Faso, 2011–2014

Variables	Numbers
*Age*	
15–20 years old	16
21–25 years old	20
*Sex*	
Female	31
Male	5
*Occupation*	
Student	14
Employed	16
Unemployed	5
*Marital status*	
Single	32
Married	4
*Living condition*	
Living alone	3
Living with relatives	33
Total participants	36

Burkina Faso 2011–2014

Ethical approval was obtained from the Burkina Faso’s National Ethics Committee for Research on Health. Written and oral informed consents were obtained from all informants. For participants under 18, we enforced the emancipated minor’s principles which waives parental consent for situation were adolescents are married or pregnant. Enforcing this principle helped us protect the women and ensure their confidentiality given that they did not want their relatives to discover that they were treated for abortion.

### Analytic approach

**Fig. 1:**
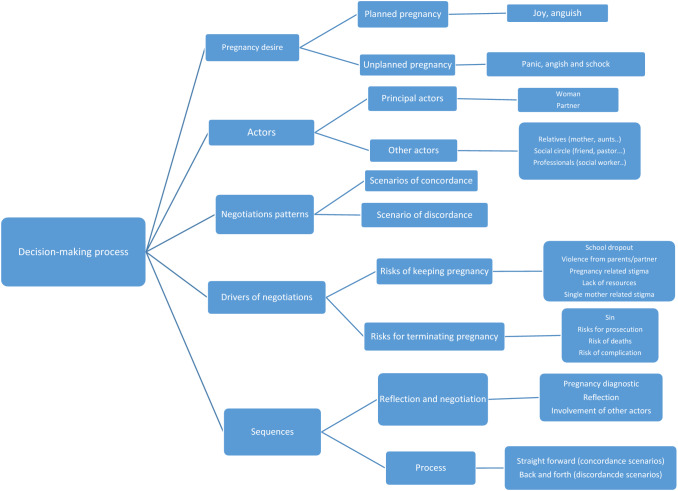
Coding tree of the decision-making process Burkina Faso, 2011–2014

Instead of viewing decision-making as an isolated act, we employed the theory proposed by Sfez (1992) of “l’homme aleatoire”(unpredictable humanity) to guide our data analysis. Based on the multi-rationality of the decision-making process, this conception takes into account interactions between individuals, as well as the influence of rationalities on each other. Audio files were transcribed and translated into French (for interviews in Moore and Dioula). We used inductive content analysis by reading through the transcripts and observation’s notes—to capture and highlight the different streams and rationales. We then coded all transcripts based on recurrent themes that we identified as key themes as described in Fig. 1. Sequences, drivers, motivations/rationales (perceived consequences of keeping pregnancy or aborting, desirability of consequences, etc.), actors and scenarios of negotiation were identified from the data coded that we regroup into two main categories. These two categories are presented here and discussed with the existing literature.

## Results

**Fig. 2:**
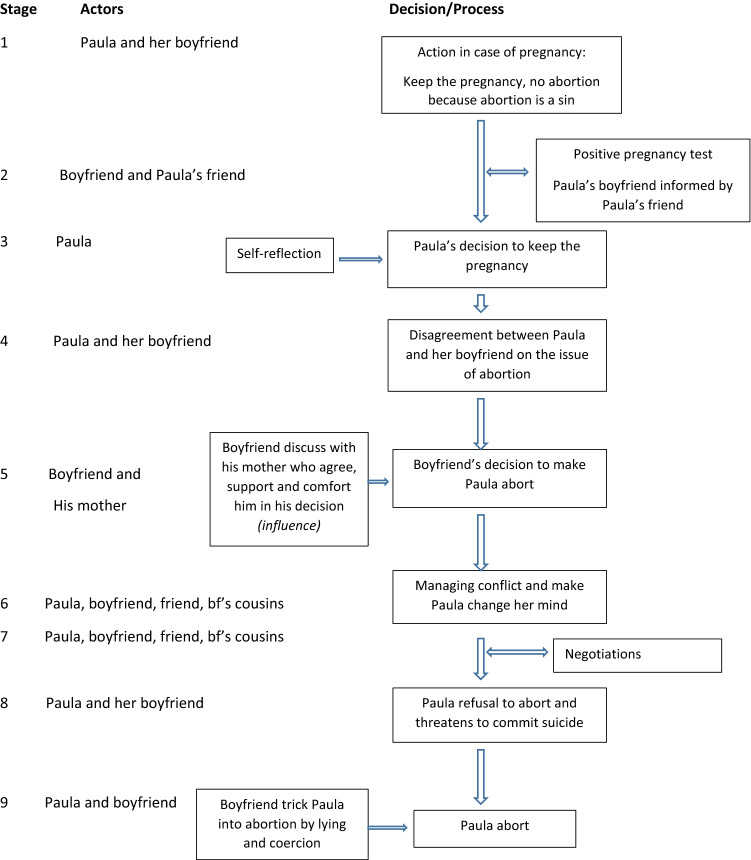
Illustration of a decision-making process, Paula, 23 year old, Burkina Faso, 2011–2014

The findings show different stages in the decision-making process. An example of decision-making process that lasted 9 weeks and drawn from Paula’s case, a 23-year-old unmarried woman who worked as a waitress in an ice cream shop, is highlighted in Fig. 2.

### Circumstances of conception and reactions to pregnancy

The abortion decision-making process includes the conditions under which the pregnancy occurs. Fertility desires and their timing, while not necessarily predictive, still very much guide the decision-making process. Some women intentionally did not use contraception because they were seeking pregnancies hoping they might resolve precarious social or economic situation. For example, Awa, a 25-year-old single mother deliberately chose to be pregnant:

I was tired of the pressure at home, people were treating me as if not getting married was my fault (…). The guy who was dating seemed serious, so I decided to be pregnant to accelerate things to escape from this nightmare.

Nine other women in our sample said that they deliberately sought to get pregnant. Sometimes called “trap pregnancies,” they can result from strategies to deal with various social constraints such as socioeconomic precarity or familial pressure to get married.

Other women we spoke to shared that they did not want to get pregnant but had not been using any form of modern contraception. Among our respondents, 20 fit into this camp, which includes women who were relying to some extent on periodic abstinence to prevent pregnancy. This was the case for Paula, early mentioned. She was using the calendar method, but her boyfriend forced her into non-consensual sex during her fertile period.

He wanted us to have sex, I told him no, that I was convinced that I was in my ‘bad period’, that he should abstain. He refused, and he forced me. His parents were asleep, so when I shouted, they didn’t hear.

Other women wanted to use modern contraception to prevent pregnancy, but had trouble accessing methods.

We have school every day, and when I am done at school, the health facilities are already closed, they will ask you to come the next day. I could not get my injection for three month” (female, 18-year-old, single, student).

Two of the women we interviewed did not want to become pregnant and were using a method of modern contraception to prevent the pregnancy. Martha, a 23-year-old student told us that she did everything she could to avoid a pregnancy:

When I discovered that I was pregnant I totally panicked, I saw my world collapsing. The thing that we did in private and I took all precautions to avoid becoming pregnant, now I find myself involved. I saw myself being judged by my entourage. I saw my studies being abandoned. Indeed, I felt the distress any human being would feel when he or she learns of the death of a loved one.

Martha’s extreme anguish at her pregnancy is palpable, and in sharp contrast to Awa’s joy that her “trap” pregnancy had worked. And yet, despite these vastly different initial reactions to their pregnancies, both women ended up seeking abortion services and justifying it by the fact that they were not ready.

I finally understood that I wasn’t ready to keep this pregnancy. I played a game and lost, he [boyfriend] refused the pregnancy. I knew that having a pregnancy is a blessing, but you need to be prepared before that, and I wasn’t prepared” (Awa, 25-year-old, single, unemployed).

### Actors and the negotiation patterns

Depending on the trajectory in which it occurs, pregnancy can strengthen or weaken the woman’s relationships with her partner, family and social circle. In response to these potential effects, the woman and the other actors often begin negotiations to decide whether to keep or end the pregnancy, knowing that either choice is significant in a context where reproductive choices are constantly negotiated between social norms and personal goals.

The decision-making process starts with the woman herself, as she is the first to know about the pregnancy. The discovery (or suspicion) of the pregnancy is accompanied by a phase of personal reflection during which the young woman weighs the risks of premarital pregnancy and the risks of inducing an abortion. This reflection can last from a few hours to several days or even weeks. With Paula, the personal reflection process took over a month. When she first learned about the pregnancy, she decided she wanted to keep it and then faced considerable opposition from her boyfriend who ultimately forced her to have an abortion.

Apart from my job, I had no other fear because 1) I was already taking care of my needs, I don’t depend on my parents to fear that they may stop supporting me or chase me. If I was able to keep my job, I would have been fine; 2) since my boyfriend had forced me to have sex, I though he couldn’t deny his responsibility for the pregnancy, maybe I will even engage (…). For me abortion was a no, I am a Christian, for us abortion is a sin, it mean killing someone. Also I did not want to take any risk of dying or being caught by police.

Thus, after a lengthy deliberation, Paula concluded that she should keep the pregnancy.

While Paula almost shared the information about her pregnancy with people around her, many others chose to keep it secret for the duration of this reflection period. One of our respondents, for instance, hesitated for a long time before sharing information with others, including her partner:

At first, I didn’t dare talk about it with anyone, even my boyfriend, I didn’t want to tell him because I was thinking he would abandon me. I’ve been thinking for several days, how am I going to do that [tell him]? Finally, I decided that I had to talk to my boyfriend about it since he is also affected (female, single, 20 years old, hairdresser).

Women shared that this time of reflection enables them to decide what they want before the personal decision becomes subject to community perspectives (both supportive and in opposition to the decision).

Actors from the women’s family and social circles who may become involved in the decision-making process include partners, mothers, sisters, fathers, colleagues, neighbors and friends. People from outside of the woman’s social network such as health workers, religious clerics and social workers may also be involved. Beside her own situation, one of our respondents explained how she participated in her friend’s decision-making process. She took that friend to a protestant priestess to help her with prayer in the effort to convince her not to have an abortion.

When we went to the priestess, she prayed and she said that her prayers show that the girl’s relative want to kill and did witchcraft to make the pregnancy happens. She said if the girl tries to have an abortion, she will either die or never have a child again. She advised her to give her parents’ number, that she will talk to them (female, 22-year-old, hairdresser).

All the 31 young women interviewed had sought or received advice from other people. The outside feedback they received could serve to either strengthen their initial choice or cause them to reconsider. The arguments used by these relatives or friends to influence the decision of keeping the pregnancy for instance included threat of denunciation to police. Eve, a 18-year-old school dropout girl, decided to keep her pregnancy after her boyfriend’s relatives threatened to report her to the police if she attempts abortion.

When my boyfriend family took me to the police, they kept the woman [abortion provider], they wanted to keep me also, but given that the pregnancy was still there, they released me. When we arrived home, my boyfriend’s father and his sisters told me that if I try anything again they will inform the police. I got scared, so I decided to just keep the pregnancy. Thank god, the same evening the pregnancy came out, while I was still with them.

The intimate partner often plays a key role in the decision-making process, and their decisions are also made through personal reflection. For instance, Mike, a 24-year-old man, panicked when his girlfriend announced her pregnancy,

I was not expecting this at all, because every time it was her fertile period, we either avoid sex, or I do withdrawal. So imagine the shock when she told me that she was pregnant. Imagine, I just graduated, I have been applying for job in vain. And I was living with my parents! How was I going to explain them this?

Given his economic dependence on his parents and the fact that pregnancy would mean living together with a woman with whom he was not sure he wanted to build a family, he concluded that he was not ready to be a father and that his partner should get an abortion.

The varying positions toward the pregnancy that a woman and her partner may have are roughly summarized in Table 2.

**Table 2:** Concordance of Abortion Desires in an Intimate Partnership, Burkina Faso, 2011–2014

		Woman	
		Wants to pursue abortion	Wants to keep the pregnancy
Woman’s partner	Wants to pursue abortion	A	B
	Wants to keep the pregnancy	C	D

Scenarios A and D depict the concordance between the desires of the woman and her intimate partner, with A representing a shared desire to pursue abortion and D representing a shared desire to keep the pregnancy. Scenarios B and C represent discordance in the couple’s abortion desires, with B representing a woman who wishes to keep the pregnancy while her partner wants her to pursue abortion and C representing a woman who wishes to pursue abortion while her partner wants her to keep the pregnancy. Scenario D, in which neither party wishes to pursue an abortion, falls outside of the scope of this paper, while six women interviewed fall into scenario A, 20 fall into scenario B, and five fall into scenario C.

Perhaps unsurprisingly, among the scenarios explored and examined in this study, the decision making was smoothest for couples falling into scenario A, where both partners agree to pursue abortion. Even if they do not share identical reasons for choosing abortion, their rationales were often complementary, leading to very little conflict. For example, Noura, a 22-year-old student living with her parents told us:

When I informed him [her partner], he asked me what I want to do? I said really, considering my situation, I don’t feel ready to have a child. He told me that he too agrees because he will not be able to take care of the child and me properly. So this was a very straightforward decision for us, we both knew we didn’t want to make it

Scenarios B and C, in contrast, tend to include a fair amount of conflict, often mediated by prolonged negotiations between the partners. These negotiations sometimes result in eventual agreement, but can also end with one partner making the final decision on his/her own. Nadia, a 23-year-old student in a professional training school, had decided immediately after she discovered her pregnancy to have an abortion and had to negotiate for days with her boyfriend who wanted her to keep the pregnancy:

When I opened the test and saw that it was positive, I called him, he came at the same time. I was upset, he told me not to worry that everything was going to be fine, that he doesn’t earn much money, but what he earns will allow us to manage the pregnancy […] When I left him I went straight to my friend’s to say that it is positive, that my boyfriend wants me to keep but I want to have an abortion. She agreed that I should have an abortion and she already knew a good method to have a safe abortion […] When I explained to him [her boyfriend] that I was going to have an abortion, he didn’t want it. […] But then, as we were talking, he calmed down a bit, he changed his mind.

If Nadia was able to convince her boyfriend and get his approval, Tenin, a 25-year-old bar waitress, ended up pursuing abortion without her partner’s consent.

It’s true that he was ready to assume the pregnancy even though he already had a wife, but I didn’t want a child now. That day I told him this child I can’t keep. He told me he doesn’t want me to have an abortion. I said okay and I didn’t talk anymore. He is talking because I told him the truth, isn’t it? If I do it in secret, can he still talk?

In the face of deadlock between partners, economic and social capital determine the outcome of the negotiation through the use of arguments, flattery, threats, blackmail and/or physical aggression. Two respondents explained:

My boyfriend started threatening me, he sent his friends to talk to me, they cajoled me, threatened me, and tried other things. I didn’t change my position. Then, his friends stopped talking to me, even my friend stopped talking to me. (Paula, 23-year-old, single, waitress)

He used many arguments to convince me to keep the pregnancy, he even promised to buy me a motorbike, I kept refusing. That’s now he started telling me that if I insist to abort, he will inform the police. I got scared and I told him that I will keep. I waited two to 3 weeks, I did my thing and told him that it was a miscarriage (laugh) (21-year-old, single, tailor)

When Paula nevertheless continued to refuse abortion, her boyfriend decided to use deception and violence to obtain the outcome he sought:

One day he came to tell me that he wanted me to come with him to go shopping. I didn’t thought that it was a trap because he had stopped talking about abortion a few days earlier. When we pulled up to a clinic, I started asking him questions, but he didn’t say anything. He asked me to wait while he went to talk to someone. Afterward, he came back with a man who told me to follow him. I asked my boyfriend why. He just told me to follow the man. When we entered a room, he told me to undress, I exclaimed “Wai! I will not undress!” When I said that, he shouted at me to remove my underwear and lie down. I was suddenly afraid, in the name of God, I was afraid of that man. I then started to cry. I removed my wrapper and my underwear and laid down. He told me to hurry because he didn’t have time. If you saw the man, he was really heavy. He pulled my legs apart, took an iron object and pushed it inside me. I started having severe pain in the lower back.

During our health facility observations, we observed the case of a young woman being admitted upon police request for an “assault” during pregnancy. She told us that she was beaten by her partner because she refused to terminate her pregnancy. One of the young men interviewed explained to us how he managed to get his partner to admit that “she was not ready and must have an abortion”:

I negotiated very hard, I confess that at some point I was forced to threaten her a little bit, even blackmail her a little bit, that I will stop the relationship if she doesn’t have an abortion. (20-year-old. student)

In these conflicting situations, when the young women concerned choose to acquiesce to their partner’s decision, it was out of a desire to save the relationship, because she was exhausted by long quarrels, and because she wanted to stop episodes of violence. In other cases, the young woman ended up agreeing to an unwanted abortion because her partner denied his responsibility for the pregnancy or simply disappeared from her life altogether.

I went to bed at night, I couldn’t sleep. What am I going to do? Give birth to a fatherless child and then say what? I want a child, but I want a child with a father. It’s true that I wanted this pregnancy, but now I can’t have it anyhow, I am not ready yet to keep it (Female, 18-year-old, single, maid)

In these cases, many women saw abortion as the only alternative to escape economic and social precarity, to avoid the social stigma of unmarried parenthood or to optimize her chances of building a future romantic relationship that conforms to social norms.

This decision-making process was, in many cases, a prolonged and disjointed, lasting anywhere from a few days to several weeks or even months. In our sample, the shortest duration was 1 day, and the longest lasted 5 months. These more prolonged processes are common in scenarios of conflict where the actors attempt to hold on as long as possible in the hopes of seeing the other give up. The longest processes are also disjointed and can happen in several stages. During this time, the process can go back and forth between abortion attempts and pursuit of the decision-making process.

## Discussion

When a young woman states “I was not ready” to justify her abortion, this expression is similar to a “Russian doll” which once opened, highlights an overlapping of personal, economic and social motivations. The young women have to weigh two types of risks and make a decision (Furby and Beyth-Marom 1992). The risks of keeping a pregnancy include the social risks of divulging premarital sexual relations, as well as the costs of parenthood on their already challenging personal, relational and social lives (Rossier et al. 2013; Chae et al. 2017; Bréda et al. 2013; Calvès et al. 2019; Ouédraogo and Guillaume 2017; Ouattara and Storeng 2008). The risks of getting an abortion can be social—because of the stigma related to the perception of abortion as a crime and a sin—medical (the risk of death and complications) and legal (risks of prosecution). Thinking through these two risks enables the young woman to “name the fetus” and decide whether to keep it or postpone motherhood (Boltanski 2004).

Combining women’s and men’s views, this study shows the important role of both the woman and her partner, as well as the family and friends, in the abortion decision-making process, as described in other studies (Kumi-Kyereme et al. 2014; Ekstrand et al. 2009). In Burkina Faso where the woman is not granted abortion decision-making rights by the government, intimate partners can be deeply involved in the abortion decision making, using a range of methods from arguing to emotional blackmail to overt force. They can be the driving force behind the decision to terminate a pregnancy (by denying it), or their decision-making power can rival and even surpass that of the woman, by deciding and implementing it. This study thus provides an insight into the manner in which power relations between men/women and elders/cadets play out in the decision-making process and the outcomes. The case of abortion is unique and important and shows how a decision commonly perceived as individual and personal is mediated by the same powerful social forces that touch other parts of life.

The limitations of this study include potential selection bias. Although we took steps to vary the respondents’ profiles, using health facilities as recruitment sites may explain the high proportion of women in precarious situations with limited power to decide on their reproductive choices in our sample. Indeed, evidence shows that most women admitted in public health facilities for abortion complications are those who lacked sufficient resources to afford safe abortion services.

### Conclusion

In the context of Ouagadougou, characterized by the restriction of abortion and stigmatization of the practice, by the complexity of gender and the generational relationships and by conflicting fertility intentions (Rossier et al. 2013; Ouédraogo and Guillaume 2017), abortion decision making should be considered in a collective dimension. The sequences show a plurality of actors engaged in a nonlinear process, which fluctuates from one choice to another according to the rationales and challenges in place. Therefore, it operates in an uncertain time frame and an unofficial social environment and has a unpredictable collaborative mechanism (Brassac and Fixmer 2004). The decision-making process is, in fact, similar to an “arena” where actors’ logics and rationales are intertwined, and which gives rise to negotiations or conflicts that can lengthen the decision-making process and expose young women to unsafe abortions.

This research provides interesting insights to guide interventions targeting unsafe abortion drivers. The findings can inform advocacy for more a liberal environment to smooth the decision-making process and reduce the power of some of the actors involved in the process. At the community level where more can be done, it can inform interventions to raise awareness on the various constraints leading to premarital pregnancies and men’s role in the abortion process to reduce mitigate the gendered vulnerability experienced by women.
